# Prognostic study of continuous variables (white blood cell count, peripheral blast cell count, haemoglobin level, platelet count and age) in childhood acute lymphoblastic leukaemia. Analysis of a population of 1545 children treated by the French Acute Lymphoblastic Leukaemia Group (FRALLE)

**DOI:** 10.1054/bjoc.2000.1504

**Published:** 2000-12

**Authors:** J Donadieu, M-F Auclerc, A Baruchel, Y Perel, P Bordigoni, J Landman-Parker, T Leblanc, G Cornu, D Sommelet, G Leverger, G Schaison, C Hill

**Affiliations:** Département de Biostatistique et d'épidémiologie, Institut Gustave Roussy, rue Camille Desmoulins, Villejuif, 94800, France; Service d'Hémato-Oncologie Pédiatrique;, Hôpital Trousseau, Paris, France; Service d'Hématologie Pédiatrique, Hôpital Saint Louis, Paris, France; Unité d'Hémato-Oncologie Pédiatrique, Hôpital Pellegrin, Bordeaux, France; Service de Médecine infantile II, Hôpital De Brabois, Vandoeuvre, France; Service d'Hématologie Pédiatrique, Clinique Universitaire Saint Luc, Bruxelles, Belgique

**Keywords:** acute lymphoblastic leukaemia, childhood, prognostic factors, continuous variable, cutpoint

## Abstract

Many cutpoints have been proposed to categorize continuous variables in childhood acute lymphoblastic leukaemia (white blood cell count, peripheral blast cell count, haemoglobin level, platelet count and age), and have been used to define therapeutic subgroups. This variation in the choice of cutpoints leads to a bias called the ‘Will Rogers phenomenon’. The aim of this study was to analyse variations in the relative risk of relapse or death as a function of continuous prognostic variables in childhood ALL and to discuss the choice of cutpoints. We studied a population of 1545 children with ALL enrolled in three consecutive protocols named FRALLE 83, FRALLE 87 and FRALLE 89. We estimated the risk of relapse or death associated with different values of each continuous prognostic variable by dividing the sample into quintiles of the distribution of the variables. As regards age, a category of children under 1 year of age was distinguished and the rest of the population was divided into quintiles. The floated variance method was used to calculate the confidence interval of each relative risk, including the reference category. The relation between the quantitative prognostic factors and the risk was monotonic for each variable, except for age. For the white blood cell count (WBC), the relation is log linear. The risk associated with WBC values in the upper quintile was 1.9 times higher than that in the lower quintile. The peripheral blast cell count correlated strongly with WBC (correlation coefficient: 0.99). The risk increased with the haemoglobin level, and the risk in the upper quintile was 1.3 times higher than that in the lower quintile. The risk decreased as the platelet count increased: the risk in the lower quintile was 1.2 times higher than that in the upper quintile. The risk increased gradually with increasing age above one year. The small subgroup of patients (2.5% of the population) under 1 year of age at diagnosis had a risk 2.6 times higher than the reference category of patients between 3 and 4.3 years of age. When the risk associated with a quantitative prognostic factor varies monotonously, the selection of a cutpoint is arbitrary and represents a loss of information. Despite this loss of information, such arbitrary categorization may be necessary to define therapeutic stratification. In that case, consensus cutpoints must be defined if one wants to avoid the Will Rogers phenomenon. The cutpoints proposed by the Rome workshop and the NCI are arbitrary, but may represent an acceptable convention. © 2000 Cancer Research Campaign http://www.bjcancer.com


British Journal of Cancer (2000) 83(12), 1617–1622
© 2000 Cancer Research Campaign
doi: 10.1054/ bjoc.2000.1504 available online at http://www.idealibrary.com on IDEAL®

http://www.bjcancer.com

### Prognostic study of continuous variables (white blood cell count, peripheral blast cell count, haemoglobin level, platelet count and age) in childhood acute lymphoblastic leukaemia. Analysis of a population of 1545 children treated by the French Acute Lymphoblastic Leukaemia Group (FRALLE)

**J Donadieu[1,2], M-F Auclerc[3], A Baruchel[3], Y Perel[4], P Bordigoni[5], J Landman-Parker[2], T Leblanc[3], G Cornu[6], D Sommelet[5], G Leverger[2], G Schaison[3] and C Hill[1] for the FRALLE group***

[1]Département de Biostatistique et d'épidémiologie, Institut Gustave Roussy, rue Camille Desmoulins, Villejuif, 94800 France; [2]Service d'Hémato-Oncologie Pédiatrique; Hôpital Trousseau, Paris, France; [3]Service d'Hématologie Pédiatrique, Hôpital Saint Louis, Paris, France; [4]Unité d'Hémato-Oncologie Pédiatrique, Hôpital Pellegrin, Bordeaux, France; [5]Service de Médecine infantile II, Hôpital De Brabois, Vandoeuvre, France; [6]Service d'Hématologie Pédiatrique, Clinique Universitaire Saint Luc, Bruxelles, Belgique

**Summary** Many cutpoints have been proposed to categorize continuous variables in childhood acute lymphoblastic leukaemia (white blood cell count, peripheral blast cell count, haemoglobin level, platelet count and age), and have been used to define therapeutic subgroups. This variation in the choice of cutpoints leads to a bias called the 'Will Rogers phenomenon'. The aim of this study was to analyse variations in the relative risk of relapse or death as a function of continuous prognostic variables in childhood ALL and to discuss the choice of cutpoints. We studied a population of 1545 children with ALL enrolled in three consecutive protocols named FRALLE 83, FRALLE 87 and FRALLE 89. We estimated the risk of relapse or death associated with different values of each continuous prognostic variable by dividing the sample into quintiles of the distribution of the variables. As regards age, a category of children under 1 year of age was distinguished and the rest of the population was divided into quintiles. The floated variance method was used to calculate the confidence interval of each relative risk, including the reference category. The relation between the quantitative prognostic factors and the risk was monotonic for each variable, except for age. For the white blood cell count (WBC), the relation is log linear. The risk associated with WBC values in the upper quintile was 1.9 times higher than that in the lower quintile. The peripheral blast cell count correlated strongly with WBC (correlation coefficient: 0.99). The risk increased with the haemoglobin level, and the risk in the upper quintile was 1.3 times higher than that in the lower quintile. The risk decreased as the platelet count increased: the risk in the lower quintile was 1.2 times higher than that in the upper quintile. The risk increased gradually with increasing age above one year. The small subgroup of patients (2.5% of the population) under 1 year of age at diagnosis had a risk 2.6 times higher than the reference category of patients between 3 and 4.3 years of age. When the risk associated with a quantitative prognostic factor varies monotonously, the selection of a cutpoint is arbitrary and represents a loss of information. Despite this loss of information, such arbitrary categorization may be necessary to define therapeutic stratification. In that case, consensus cutpoints must be defined if one wants to avoid the Will Rogers phenomenon. The cutpoints proposed by the Rome workshop and the NCI are arbitrary, but may represent an acceptable convention. © 2000 Cancer Research Campaign http://www.bjcancer.com

**Keywords**: acute lymphoblastic leukaemia; childhood; prognostic factors; continuous variable; cutpoint

Prognostic factors can be dichotomous by nature, but are more often either polytomous or continuous. Continuous variables are almost always used in prognostic models after categorization. Categorizing a continuous variable has adverse consequences: it requires the definition of cutpoints and implies a threshold effect: the patients immediately above and below the cut off value are supposed to be less similar than the patients at each end of the same category. Choosing a single cutpoint will result in a loss of statistical power in most instances. But many practical or bio logical reasons lead researchers to use one or several cutpoints in order to define the therapeutic classification. Different methods have been proposed to select cutpoints. One is called the optimal cutpoint method (Hilsenbeck et al, 1992). It is based on the *P* value of repeated univariate analyses of the same sample. This method has been rejected (Hill, 1993; Altman et al, 1994) because it artificially decreases the *P* value. Another possibility is to divide the sample into equal parts (thirds or quintiles for instance) on the basis of the continuous variable under study, and to evaluate the risk in each category.

Quantitative prognostic variables in childhood ALL include the white blood cell count, peripheral blast cell count, haemoglobin level, platelet count and age at diagnosis. The prognostic value of these variables was identified more than 30 years ago (Tivey,

*Received 17 February 2000*
*Revised 9 August 2000*
*Accepted 15 August 2000*

*Correspondence to:* J Donadieu

* Members of the FRALLE group are listed at the end of the paper.

1952; Zuelzer, 1964) and they remain prognostic today despite improvements in treatment (Reiter et al, 1994). The quantitative nature of these variables necessitates the use of cutpoints to define prognostic subgroups and to select a treatment (Mastrangelo, 1986a; Bleyer, 1989). Sometimes a single cutpoint is chosen, but in other studies several cutpoints are selected, usually in an arbitrary manner. The hypotheses underlying the choice of cutpoints are rarely explained in the literature or discussed. Table 1 shows examples of the different cutpoints for age and white blood cell counts chosen by different teams in prognostic studies. The wide diversity of cutpoints can have significant consequences and makes it difficult to compare the results of different protocols. The same patient can be considered as having a low risk or a high risk depending on the prognostic classification used. Such differences in classification may lead to an apparent improvement in the outcome of all the different prognostic risk groups, despite the lack of any global improvement. This is called the Will Rogers phenomenon (Feinstein et al, 1985). The purpose of this study, using a published data set (Donadieu et al, 1998), was to explore systematically the relationship between continuous prognostic variables and outcome in childhood ALL.

### MATERIALS AND METHODS

### French acute lymphoblastic leukaemia group (FRALLE) 83, 87 and 89 protocols

FRALLE 83 (May 1983–September 1986) included 818 patients from 59 centres, FRALLE 87 (June 1987–March 1989) 361 patients from 37 centres, and FRALLE 89 (April 1989–January 1992) 586 patients from 35 centres. A brief description of the three protocols can be found in Table 2. The results have been published elsewhere (Donadieu et al, 1998).

### Exclusion from the study

Of the 1766 patients included in the three protocols, 221 were excluded from the present analysis: 3 for errors in the initial diagnosis (AML or ALL 3), 21 because they had mediastinal T lymphoma without bone marrow infiltration, 67 because they did not live in France, 112 because no baseline data were available (109 in FRALLE 83, 2 in FRALLE 87 and 1 in FRALLE 89), 9 because their centres failed to provide more than half the baseline and follow-up data, and two because of constitutional trisomy 21. 7 patients were excluded because at least one of the variables studied was missing.

**Table 2** Description of the FRALLE 83, 87 and 89 protocols

All three protocols included an induction, a consolidation and a maintenance phase. All patients with ALL and age 0 to 20 years were included. In each protocol, the treatment was adapted to the level of risk by defining low, average and high risk groups. The definition of the risk groups was different in the different protocols, the low risk group being larger in FRALLE 87 and 89 than in FRALLE 83.

FRALLE 83 protocol compared Vincristine to Vindesine in all patients, compared standard to intensive consolidation in the intermediate risk group, and compared testicular irradiation to no irradiation for boys in the high risk group.

FRALLE 87 and 89 protocols compared Daunorubicine to Rubidazone in all patients, and compared 3 to 8 g/m$^2$ of methotrexate in the low-risk group. In the FRALLE 89 protocol, the dose of folinic acid was reduced to 12 pulses of 15 mg/m$^2$.

The detailed protocols have been published elsewhere (Donadieu et al, 1998).

### Endpoints, cut-off dates for analysis, and follow-up

The chosen endpoint was primary failure, toxic death or relapse at any site. Event-free survival (EFS) was defined as the time between the diagnosis and the time of the event or the time of the last examination when no event occurred. If a patient failed to enter complete remission, the event was recorded as occurring immediately, and EFS was 0. The cut-off date for the analysis was September 30, 1999.

### Study variables

We studied quantitative variables with a known prognostic value recorded in the FRALLE data base, i.e. age at diagnosis and the complete blood count. If several blood counts were available at initial diagnosis, we used the lowest haemoglobin level and platelet count before any transfusion, and the highest white blood cell count and peripheral blast cell count.

**Table 1** Number and values of cutpoints for age and white blood cell counts (WBC) used in the literature to define prognostic categories in ALL

| | Age cutpoints | | WBC cutpoints | |
|---|---|---|---|---|
| Cooperative institution (reference) | Number | Values in years | Number | Values in 1000/mm$^3$ |
| French group (Jacquillat et al, 1978) | 3 | 1–15–20 | 1 | 35 |
| FRALLE 83 (Schaison et al, 1990) | 2 | 2–10 | 3 | 15–50–100 |
| NOPHO group (Gustafsson et al, 1998) | 1 | 1–2–10 | 4 | 10–20–50–100 |
| Japanese group (Hiyoshi et al, 1985) | 4 | 1–4–6–10 | 4 | 5–10–50–200 |
| CCG 1972–1975 (Robinson et al, 1980) | 5 | 2–4–6–8–10 | 5 | 5–10–20–50–100 |
| CCG 160 (Bleyer et al, 1986) | 4 | 1–3–6–10 | 2 | 20–50 |
| BFM before 1981 (Bucsky et al, 1988) | 5 | 0.5–1–1.5–2–10 | | NA |
| BFM 81 (Schrappe et al, 1987) | 4 | 1–2–10–14 | 3 | 10–50–100 |
| BFM 86 (Reiter et al, 1994) | 3 | 1–6–10 | 4 | 10–20–50–200 |
| DFCI 85–01 (Schorin et al, 1994) | 3 | 1–2–9 | 3 | 20–50–100 |
| UKALL X (Chessells et al, 1995) | 2 | 1–10 | 4 | 10–20–50–100 |

Each cutpoint is included in the interval to the right, for instance cutpoint 1 and 10 for age define the intervals age <1, 1 to 9 and 10+.
NA: Not available. WBC is indirectly in the model, which included 0.2 Log (peripheral blast cell count +1).

 © 2000 Cancer Research Campaign

### Statistical methods

BMDP® software was used for all statistical analyses. Median follow-up was estimated from a Kaplan–Meier curve in which the last follow-up date was used for the patients who survived as if it was an endpoint, and in which the other patients were censored at the time of death (Schemper and Smith, 1996). The actuarial method was used to estimate survival rates. A Cox semi-parametric model was used to estimate the hazard ratio in each category (Cox, 1972; Collet, 1994). The cutpoints were obtained by dividing the overall sample into quintiles. A category below 1 year of age corresponding to 39 patients (2.5% of the population) was defined, as proposed in the literature (Mastrangelo, 1986b; Smith et al, 1996), and the rest of the population was divided into quintiles. For each cutpoint, the boundary value was included in the higher category. The variance of the hazard ratios was calculated by the floated variance method (Easton et al, 1991), in order to attribute some imprecision to the reference category. In the figures, the hazard ratio for each category (after log transformation for white blood cells) is plotted at the median value of the category on the x axis. Another Cox regression model estimated the trend in the log hazard ratios.

### RESULTS

The median follow-up was 111 months in FRALLE 83, 106 months in FRALLE 87, and 86 months in FRALLE 89. Among the 1545 patients, a total of 781 events were recorded during the study period, corresponding to 36 toxic deaths during induction, 42 induction failures, 65 toxic deaths after induction (patients in first remission) and 638 relapses. Complete remissions were obtained after induction in 1467 patients (95%), while 78 patients (5%) failed to enter complete remission after the induction phase.

The 10-year EFS rate was 48.2% (SE 1.3), and the 10-year survival rate was 61.4% (SE 1.3).

### Age

The distribution of age was approximately log-normal, with a median of 5.1 years. The risk of events varied with age according to a U-shaped curve. The group of patients under one year of age had a risk 2.6 times higher than that of patients between 3 and 4.3 years of age (the reference quintile). A Cox regression model with a specific hazard ratio for the population under one year of age and a linear trend in the older population was fitted, leading to the estimation:

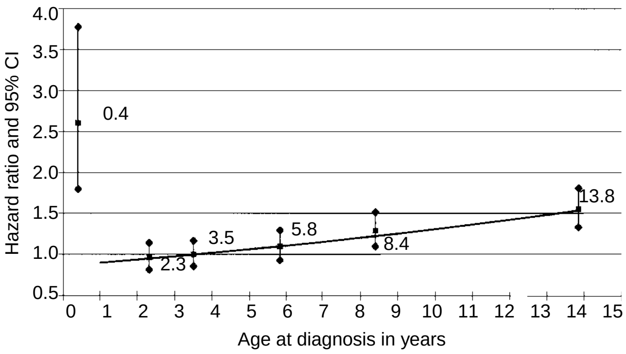

**Figure 1** Hazard ratio for relapse or death and 95% confidence interval for age at diagnosis under 1 year and for each quintile of age at diagnosis above 1 year (using the median value of the quintile on x axis)

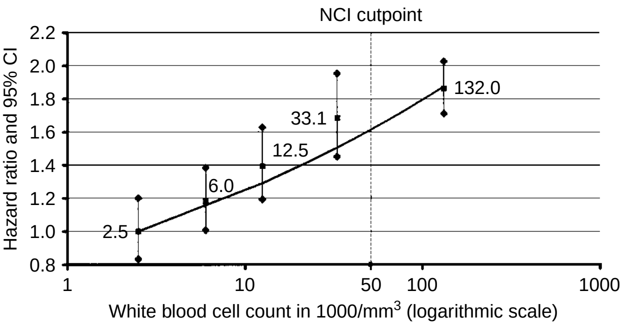

**Figure 2** Hazard ratio for relapse or death and 95% confidence interval for each quintile of white blood cell count at diagnosis (using the median value of the quintile on x axis)

$$HR = \exp\,[1.09_{(\text{if age} < 1\text{ year})} + 0.042 * (\text{age}-3.5)_{(\text{if age} \geq 1\text{ year})}]$$

The risk in the highest quintile was 1.6 times higher than that in the reference quintile Figure 1).

### White blood cell count

The white blood cell count (WBC) had an exponential distribution with a median of 12 000/mm$^3$. The risk of relapse or toxic death increased with increasing WBC (Figure 2). A Cox regression model was fitted with a linear effect of log (WBC):

$$HR = \exp\,[0.158 * (\text{Log}\,(WBC + 0.1) - \text{Log}\,2.5)]$$

where WBC is expressed in 1000/mm$^3$. The risk in the highest quintile was 1.9 times higher than that in the lowest quintile.

### Peripheral blast cell count

Peripheral blast cells represent a large proportion of white cells at diagnosis, and their numbers correlate strongly (r = 0.99). Thus, the peripheral blast cell count provides the same prognostic information as the WBC.

### Haemoglobin level

The distribution of haemoglobin levels at diagnosis was approximately normal, with a median of 7.7 g dl$^{-1}$. A Cox regression model was fitted with a linear effect of the haemoglobin level:

$$HR = \exp\,[0.037 * (Hb - 4.6)]$$

The risk in the highest quintile was 1.3 times higher than that in the lowest quintile (Figure 3).

### Platelet count

The distribution of the platelet count was log-normal, with a median of 56 000/mm$^3$. The hazard ratio of events fell when platelets increased. A Cox regression model was fitted with a linear effect of the platelet count:

$$HR = \exp\,[-\,0.0012 * (\text{Platelet}-232)].$$

The risk in the lowest quintile was 1.2 times higher than that in the highest quintile (Figure 4).

*© 2000 Cancer Research Campaign*

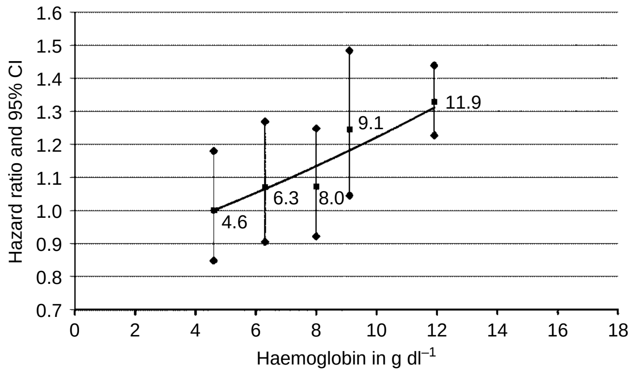

**Figure 3** Hazard ratio for relapse or death and 95% confidence interval for each quintile of haemoglobin level at diagnosis (using the median value of the quintile on x axis)

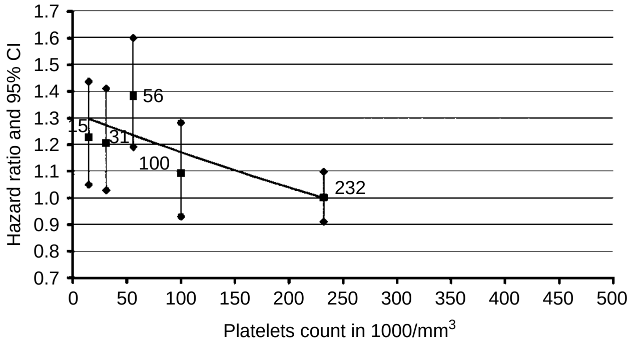

**Figure 4** Hazard ratio for relapse or death and 95% confidence interval for each quintile of platelet count at diagnosis (using the median value of the quintile on x axis)

| | Classification 1 | | |
|---|---|---|---|
| | Low risk | High risk | |
| % of patients | 33 | 67 | |
| 5-year EFS | 80% | 35% | |
| % of patients | 33 | 33 | 33 |
| 5-year EFS | 80% | 50% | 20% |
| % of patients | 67 | | 33 |
| 5-year EFS | 65% | | 20% |
| | Low risk | | High risk |
| | Classification 2 | | |

**Figure 5** The Will Rogers phenomenon: a teaching example. Note: The overall 5-year EFS in the sample is 50%. There are three subgroups of equal size, with 5-years EFS values of 80%, 50% and 20%. The definition of the 'low risk' and 'high risk' categories changes between classification 1 and 2, resulting in a shift between the categories of one third of the sample. Classification 1 leads to a better result in each category (80% and 35% for the low and high risk group) compared to classification 2 (60% and 20% respectively), without any change in the overall results

| | Classification 1 | | | | |
|---|---|---|---|---|---|
| | Low risk | Standard risk | | High risk | |
| % of patients | 25 | 51.8 | | 23.2 | |
| 5-year EFS | 63.3% | 52% | | 43.8% | |
| WBC in 1000/mm$^3$ | 0 to 4.9 | 5 to 14.9 | 15 to 49.9 | 50 to 399.9 | 400 + |
| % of patients | 25 | 30 | 22 | 21 | 2 |
| 5-year EFS | 63.3% | 55% | 48% | 45% | 34% |
| % of patients | 55 | | 43 | | 2.2 |
| 5-year EFS | 59% | | 46% | | 34% |
| | Low risk | | Standard risk | | High risk |
| | Classification 2 | | | | |

**Figure 6** The Will Rogers phenomenon: a real example with the FRALLE data set. Note: The overall 5-year EFS is 52%. The variable used to define the risk category is the white blood cell count expressed in 1000/mm$^3$. Classification 1 leads to an apparent improvement in the results for each category as compared to classification 2. Moving the WBC cutpoint from 5 to 15 moves 30% of the patients from the low-risk group to the standard risk group. At the other extreme, introducing a cutpoint of 400 identifies a small subgroup of 2% of patients with a very bad prognosis.

### DISCUSSION

The main result of our study is that, except for age, the relation between the risk of relapse or toxic death and each continuous prognostic factor in childhood ALL is monotonic. The patients under 1 year of age had a markedly increased risk, while the risk for patients above 1 year increased linearly with age. Except for age, the ratio between the risk in the worst quintile and that in the best quintile never exceeded 2.

We performed a univariate analysis for each variable dividing the sample into quintiles. This avoids the optimal cutpoint method and the associated multiple tests. We used a linear or log linear model, which is simpler than fractional polynomials (Royston and Altman, 1994; Sauerbrei, 1999).

When the risk varies monotonously as a function of a continuous variable, any categorization of the variable represents a loss of statistical information, and there is no optimal cutpoint. For instance, the selection of a cutpoint of 50 000 WBC/mm$^3$ for WBC is arbitrary, as is the selection of 9 years for age.

The analysis was not adjusted on other known prognostic variables. Here, we present an exploratory study of the relation between the risk of relapse or death and different continuous variables in childhood acute lymphoblastic leukaemia. A multivariate analysis using the same data set showed that age and the white blood cell count were the most significant prognostic variables in childhood acute lymphoblastic leukaemia, whereas the haemoglobin level and the platelet count had a lower prognostic value (Donadieu et al, 1998).

Many cutpoints have been used in the literature (Table 1) but this diversity makes it difficult to compare the results of different protocols. The same patient can be considered as having a low risk or as having a high risk depending on the prognostic classification used. Such classification changes may lead to an apparent improvement in each prognostic risk group, without any overall improvement (Feinstein et al, 1985). This is illustrated in Figure 5 with a teaching example, and in Figure 6 with our data set. A consequence is that, overall results from institutions using different classifications are comparable, whereas results within prognostic subgroups are not.

Nevertheless, prognostic subgroups are necessary for clinical decision making, since the treatment is usually adapted to the risk. To be able to compare results between institutions within a risk category, it would be better to agree upon a convention for the choice of cutpoints, however arbitrary they may be, rather than

© 2000 Cancer Research Campaign change the values of quantitative variables defining treatment groups at each new analysis or at each new protocol.

Two consensus conferences (the Rome workshop (Mastrangelo, 1986b) and the NCI meeting (Smith et al, 1996)) have proposed cutpoints for age and white blood cells, which represent the most significant continuous variables (Donadieu et al, 1998). They both proposed 1 and 9 years, and 50 000 WBC/mm$^3$ as cutpoints for age and the white blood cell count, respectively. These values are both arbitrary and acceptable.

### REFERENCES

Altman DG, Lausen B, Sauerbrei W and Schumacher M (1994) Danger of using "optimal" cutpoints in the evaluation of prognostic factors. *J Natl Cancer Inst* **86**: 829–835

Bleyer WA (1989) Remaining problems in the staging and treatment of childhood lymphoblastic leukemia. *Am J Pediatr Hematol Oncol* **11**: 371–379

Bleyer WA, Sather H, Coccia P, Lukens J, Siegel S and Hammond GD (1986) The staging of childhood acute lymphoblastic leukemia: strategies of the Childrens Cancer Study Group and a three-dimensional technic of multivariate analysis. *Med Pediatr Oncol* **14**: 271–280

Bucsky P, Reiter A, Ritter J, Dopfer R and Riehm H (1988) Die akute lymphoblastiche leukämie im Säuglingsalter: Eregebnissse aus fünf multisentrischen therapiestudien ALL-BFM 1970–1986. *Klin Pädiatr* **200**: 177–183

Chessells JM, Bailey C and Richards SM (1995) Intensification of treatment and survival in all children with lymphoblastic leukaemia: results of UK medical research council UKALL X. *Lancet* **345**: 143–148

Collet D (1994) *Modelling survival data in medical research*. Chapman and Hall: London

Cox DR (1972) Regression models and life tables (with discussion) *J R Stat Soc* **34**: 248–275

Donadieu J, Auclerc MF, Baruchel A, Leblanc T, Landman-Parker J, Perel Y, Michel G, Cornu G, Bordigoni P, Sommelet D, Leverger G, Hill C and Schaison G (1998) Critical study of prognostic factors in childhood acute lymphoblastic leukaemia: differences in outcome are poorly explained by the most significant prognostic variables. *Br J Haematol* **102**: 729–739

Easton DF, Peto J and Babiker AGAG (1991) Floating absolute risk: an alternative to relative risk in survival and case control analysis avoiding an arbitrary reference group. *Stat Med* **10**: 1025–1035.

Feinstein AR, Sosin DM and Wells CK (1985) The Will Rogers phenomenon: Stage migration and new diagnosis techniques as a source of misleading statistics for survival in cancer. *N Engl J Med* **312**: 1604–1608

Gustafsson G, Kreuger A, Clausen N, Garwicz S, Kristinsson J, Lie SO, Moe PJ, Perkkio M, Yssing M and Saarinen-Pihkala UM (1998) Intensified treatment of acute childhood lymphoblastic leukaemia has improved prognosis, especially in non-high-risk patients: the Nordic experience of 2648 patients diagnosed between 1981 and 1996. Nordic Society of Paediatric Haematology and Oncology. *Acta Paediatrica* **87**: 1151–1161

Hill C (1993) Valeur pronostique d'une variable continue et point de césure optimal. *Bull Cancer* **80**: 649–652

Hilsenbeck SM, Clark GM and McGuire WL (1992) Why do so many prognostic factors fail to pan out? *Breast Cancer Res Treat* **22**: 197–206

Hiyoshi Y, Fujimoto T, Kuriya N, Otani Y, Ibu K, Yanai M, Sasaki K, Shingaki Y, Yokoyama T and S-K (1985) Prognostic factors in children with acute lymphoblastic leukemia. Part II: multivariate analysis *Jpn J Clin Oncol* **15**: 13–23

Jacquillat C, Weil M, Auclerc MF, Chastang C, Flandrin G, Izrael V, Schaison G, Degos L, Boiron M and Bernard J (1978) Prognosis and treatment of acute lymphoblastic leukemia. *Cancer Chemother Pharmacol* **1**: 113–122

Mastrangelo R (1986a) The problem of "staging" in childhood acute lymphoblastic leukemia: a review. *Med Pediatr Oncol* **14**: 121–123

Mastrangelo R (1986b) Report and recommendations of the Rome workshop concerning poor-prognosis ALL in children: Biologic bases for staging, stratification, and treatment. *Med Pediatr Oncol* **14**: 191–194

Reiter A, Schrappe M, Ludwig WD, Hiddemann W, Sauter S, Henze G, Zimmermann M, Lampert F, Havers W, Niethammer D, Odenwald E, Ritter J, Mann G, Welte K, Gadner H and Riehm H (1994) Chemotherapy of 998 unselected childhood acute lymphoblastic leukemia patients. Results and conclusions of the multicenter trail ALL-BFM 86. *Blood* **84**: 3122–3133

Robison LL, Sather HN, Coccia PF, Nesbit ME and Hammond GD (1980) Assessment of the interrelationship of prognostic factors in childhood acute lymphoblastic leukemia. *Am J Pediatr Hematol Oncol* **2**: 5–13

Royston D and Altman DG (1994) Regression using fractional polynomials of continuous covariates: parsimonious parametric modelling. *Applied Statistics* **43**: 429–467

Sauerbrei (1999) Building multivariable prognostic and diagnostic models: transformation of the predictors by using fractional polynomials. *J R Statist Soc A* **162**: 71–94

Schaison G, Olive D, Leverger G, Vannier JP, De Lumley L, Bancillon A and Cornu G (1990) Treatment of acute lymphoblastic leukemia: protocol Fralle 83–85. *Haematol Blood Transfu* **33**: 467–472

Schemper M and Smith TL (1996) A note on quantifying follow-up in studies of failure time. *Controlled Clin Trials* **17**: 343–346

Schorin MA, Blattner S, Gelber RD, Tarbell NJ, Donnelly M, Dalton V, Cohen HJ and Sallan SE (1994) Treatment of childhood acute lymphoblastic leukemia: results of Dana-Farber Cancer Institute/Children's hospital acute lymphoblastic leukemia consortium protocol 85–01. *J Clin Oncol* **12**: 740–747

Schrappe M, Beck J, Brandeis WE, Feickert HJ, Gadner H, Graf N, Havers W, Henze G, Jobke A, Kornhuber B and et al (1987) Treatment of acute lymphoblastic leukemia in childhood and adolescence: results of the multicenter therapy study ALL-BFM 81. *Klin Pädiatr* **199**: 133–150

Smith M, Arthur D, Camitta B, Caroll AJ, Crist W, Gaynon P, Gelber R, Heerema N, Korn EL, Link M, Murphy S, Pui CH, Pullen J, Reaman G, Sallan SE, Sather H, Shuster J, Simon R, Trigg M, Tubergen D, Uckun F and Ungerleider R (1996) Uniform approach to risk-classification and treatment assignment for children with ALL. *J Clin Oncol* **14**: 18–24

Tivey H (1952) Prognosis for survival in the leukemias of childhood. *Pediatrics* **10**: 48–59

Zuelzer WW (1964) Implications of long-term survival in acute stem cell leukemia of childhood treated with composite cyclic therapy. *Blood* **24**: 477–494

*© 2000 Cancer Research Campaign*

### APPENDIX 1 LIST OF THE MEMBERS OF THE FRALLE GROUP

Pautard B, Centre Hospitalier Universitaire, Amiens; Berthou C, Centre Hospitalier Universitaire, Brest, Cornu G, Département d'Hématologie Infantile, Faculté de Médecine de Louvain, Bruxelles, Belgique; Perel Y, Centre Hospitalier Pellegrin, Bordeaux; P Boutard; Centre Hospitalier Universitaire, Caen; Demeocq F, Hotel Dieu, Centre hospitalier Universitaire, Clermont Ferrand; Lemerle S, Bernaudin F, Centre Hospitalier Inter-Communal de Créteil, Créteil; Couillaut G, Centre hospitalier Universitaire, Dijon; Bader-Meunier B, Dommergues JP, Tchernia G, Hôpital Bicétre, Le Kremlin Bicétre; Piguet C, De Lumley L, Centre hospitalier universitaire, Limoges; Thuret I, Michel G, Hôpital de la Timone, centre Hospitalier Universitaire, Marseille; Bordigoni P, Olive-Sommelet D, Schmitt C, Hopitaux de Brabois, Centre Hospitalier Universitaire, Nancy; Blanche S, Casanova JL, Debré M, Dupuis S, Fischer A, Quartier P, Thomas C, Hôpital Necker Enfants-Malades, Paris; Auclerc MF, Baruchel A, Berger R, Espérou-Bourdeau H, Leblanc T, Lepage E, Schaison G, Sigaux F, Hôpital Saint-Louis, Paris; Auvrignon A, donadieu J, El Adjidi N, Landman-Parker J, Leverger G, Rognon C, Tabone MD, Hôpital A Trousseau, Paris; Bergeron C, Edan C, Gandemer V, Le Gall E, Centre Hospitalier Universitaire, Rennes; Vannier JP, Hôpital C Nicole, Rouen; Freycon F, Stephan JL, Centre Hospitalier Universitaire, Saint Etienne; Lamagnéres JP, Lejars O, Hôpital de Clocheville, Tours; Hayat M, Pico JL; Institut Gustave Roussy, Villejuif.

*© 2000 Cancer Research Campaign*
